# Cecal Gastrointestinal Stromal Tumor Mimicking an Appendiceal Inflammatory Mass: Diagnostic Challenges and Surgical Management: A Case Report

**DOI:** 10.3390/jcm15134857

**Published:** 2026-06-23

**Authors:** Ardak Omarbekov, Kulzhan Berikkhanova, Vladimir Grigorevskii, Saken Kozhakhmetov, Leila Gassanova, Daulet Yessenbaev, Dulat Turebayev, Medet Toleubayev, Kairat Adaibaev, Zhannat Zhakiyanova

**Affiliations:** 1 Professor G.V. Tsoi Scientific and Educational Center of Surgery, Astana Medical University, Astana 010000, Kazakhstan; 2 National Laboratory Astana, Nazarbayev University, Astana 010000, Kazakhstan; 3 University Medical Center, Nazarbayev University, Astana 010000, Kazakhstan; 4 Department of Neurology, Ophthalmology and Otorhinolaryngology, Semey Medical University, Semey 071400, Kazakhstan

**Keywords:** gastrointestinal stromal tumor, cecum, appendiceal infiltrate, differential diagnosis, ileocolic anastomosis, case report

## Abstract

**Background:** Gastrointestinal stromal tumors (GISTs) are rare mesenchymal neoplasms, accounting for approximately 1–3% of all gastrointestinal tumors, with an annual incidence of 1–2 cases per 100,000 population worldwide. They arise from the interstitial cells of Cajal and are most commonly located in the stomach and small intestine. **Methods:** We report a case of a 39-year-old man admitted with a preliminary diagnosis of an appendiceal inflammatory mass with suspected abscess formation. **Results:** The patient presented with right iliac fossa pain, fever, signs of pronounced systemic intoxication and laboratory findings consistent with inflammatory syndrome. Abdominal computed tomography revealed a mass in the right iliac region with infiltration of the surrounding adipose tissue, suggestive of an appendiceal infiltrate. Emergency surgical exploration identified a tumor originating from the cecum. Radical resection of the ileocecal region with side-to-side ileo-ascending anastomosis was performed. Histopathological examination confirmed a spindle-cell variant of GIST. The postoperative course was uneventful. **Conclusions:** This case highlights the diagnostic challenges of atypically localized GISTs, which may clinically and radiologically mimic inflammatory conditions such as appendiceal infiltrate. Conventional imaging modalities may be insufficient for definitive differential diagnosis. Surgical resection remains the cornerstone of treatment, with histopathological and immunohistochemical evaluation establishing the final diagnosis. Early identification and complete tumor excision are essential for optimizing clinical outcomes and long-term prognosis. Adjuvant therapy with tyrosine kinase inhibitors should be considered based on individual recurrence risk.

## 1. Introduction

Gastrointestinal stromal tumors (GISTs) are rare mesenchymal neoplasms of the gastrointestinal tract, representing approximately 1–3% of all gastrointestinal malignancies, with an estimated annual incidence of 1–2 cases per 100,000 individuals worldwide [1,2,3]. These tumors originate from the interstitial cells of Cajal and are most frequently located in the stomach and small intestine [1,4]. GISTs constitute a distinct clinicopathological nosologic entity, that differs from other nonepithelial gastrointestinal tumors in both immunohistochemical and molecular characteristics [1,5]. The pathogenesis of most GISTs is associated with mutations in the KIT and PDGFRA genes, which have important prognostic and therapeutic implications [4,6,7]. The disease is most frequently diagnosed in adults older than 50 years, although it may also occur in younger patients [4].

The average age at diagnosis typically ranges from 40 to 70 years. Clinical manifestations are often nonspecific and commonly include abdominal pain, gastrointestinal bleeding, or nonspecific abdominal discomfort, which may mimic inflammatory or infectious conditions [1,3].

Consequently, early diagnosis remains challenging, particularly in cases with atypical tumor localization, frequently resulting in misdiagnosis and delays in treatment [1,3].

Colonic GISTs, especially those arising in the cecum, are exceedingly rare and may clinically mimic more common conditions, such as acute appendicitis or appendiceal inflammatory masses. This overlap in clinical and radiological findings often contributes to diagnostic errors. In this report, we present a case of cecal GIST in a 39-year-old patient initially diagnosed as an appendiceal inflammatory mass, highlighting the associated diagnostic challenges and surgical management considerations.

## 2. Case Presentation

### 2.1. Clinical History and Presentation

A 39-year-old male patient was admitted on an emergency basis with a suspected appendiceal inflammatory mass and possible abscess formation.

His medical history included prior treatment for a similar condition two months earlier. At that time, he had been admitted to a district hospital with a diagnosis of appendiceal infiltrate with suspected abscess formation and underwent right iliac fossa laparotomy with drainage of the appendiceal infiltrate.

However, no appendiceal abscess was identified intraoperatively. The patient was discharged on the fourth postoperative day after refusing further treatment. On the same day following discharge, the patient was readmitted to a regional hospital, where the dense appendiceal infiltrate and right-sided hydronephrosis were diagnosed.

Laboratory investigations at admission demonstrated a serum creatinine level of 146 μmol/L and a urea level of 9.2 mmol/L, findings consistent with impaired urinary outflow.

Ultrasonography of the kidneys revealed dilation of the pelvicalyceal system of the right kidney, confirming right-sided hydronephrosis.

Owing to the anatomical proximity of the right ureter to the inflammatory process, the dense appendiceal infiltrate caused extrinsic compression of the right ureter, leading to impaired urinary outflow and secondary obstructive hydronephrosis. To decompress the upper urinary tract, transurethral stenting of the right ureter was performed. The patient subsequently received comprehensive conservative treatment for the underlying condition and was discharged in satisfactory condition on the eighth day of hospitalization.

Two months later, the patient presented to the emergency department of a municipal hospital with worsening general condition, abdominal pain, fever (38 °C), chills, and weakness.

Physical examination revealed tenderness in the right iliac fossa with positive rebound tenderness.

Laboratory investigations demonstrated leukocytosis (12.3 × 10^9^/L), elevated erythrocyte sedimentation rate (ESR; 48 mm/h), and increased C-reactive protein (CRP; 17.6 mg/L). Table 1 summarizes the temporal changes in the patient’s laboratory parameters at admission, during the postoperative period, and at discharge.

A schematic timeline of the patient’s clinical course and therapeutic interventions is presented in Figure 1.

### 2.2. Imaging Findings

Abdominal ultrasonography revealed an inflammatory mass in the right iliac region with suspected abscess formation (Figure 2). Renal ultrasonography showed no pathological findings. Computed tomography (CT) of the abdomen demonstrated a mass in the right iliac region with infiltration of the surrounding adipose tissue, consistent with an appendiceal inflammatory mass (Figure 3).

Considering the clinical findings, imaging results, and in view of the inability to reliably exclude abscess formation within the inflammatory infiltrate, as well as the risk of disease progression and development of complications, a decision was made to perform surgical intervention.

Emergency lower midline laparotomy revealed a dense inflammatory–tumor-like conglomerate in the ileocecal region. A firm mass measuring approximately 8.0 × 5.0 cm was identified in the retroperitoneal space, arising from the posterior wall of the cecum and extending into the ileocecal region and adjacent retroperitoneal tissues (Figure 4). No regional lymph node involvement or distant metastases were detected.

### 2.3. Surgical Intervention

Emergency lower midline laparotomy revealed a dense adhesive inflammatory–tumor-like conglomerate in the ileocecal region. The mass measuring approximately 8.0 × 5.0 cm was identified in the retroperitoneal space, originating from the posterior wall of the cecum and extending into the ileocecal region and adjacent retroperitoneal tissues (Figure 4).

No evidence of regional lymph node involvement or distant metastases was identified.

The terminal ileum was intimately adherent to the cecal cupola. The appendix was enlarged, and demonstrated signs of phlegmonous inflammation. Dilatation of the ileum was observed proximal to the ileocecal junction due to compression by the inflammatory infiltrate. Intraoperative assessment revealed no evidence of right ureter involvement in the pathological process, with no signs of compression or invasion of the urinary tract structures.

Based on the intraoperative findings, the following diagnosis was established: cecal tumor of uncertain etiology complicated by partial intestinal obstruction and secondary phlegmonous appendicitis. The tumor was staged as cT4bN0M0. Given the intraoperative finding—namely, the presence of a dense tumor-inflammatory conglomerate in the ileocecal region, the technical impossibility of safely isolating the tumor from the surrounding tissues, signs of intestinal obstruction, and secondary inflammation of the vermiform appendix—radical removal of the pathological focus was deemed necessary. Radical resection of the ileocecal region together with the tumor-inflammatory conglomerate was performed. In accordance with oncological surgical principles, resection of the terminal ileum and the ascending colon with regional lymphadenectomy and adequate surgical margins was carried out, followed by the formation of a side-to-side ileo-ascending (ileocolic) anastomosis.

### 2.4. Histopathological Findings

Macroscopic examination revealed a dense tumor with a granular appearance, containing areas of necrosis and hemorrhage. Histological evaluation demonstrated a spindle-cell variant of GIST with infiltration into the surrounding tissues (Figure 5). Histopathological examination of the resected specimen demonstrated a gastrointestinal stromal tumor composed predominantly of intersecting fascicles of spindle-shaped neoplastic cells with focal small nodular areas formed by epithelioid-type cells.

Tumor infiltration into the surrounding adipose tissue was identified, indicating locally invasive growth. In addition, the tumor extended into multiple layers of the small intestinal wall, including the muscular layer, submucosal layer, and intermuscular layer.

These findings confirmed the infiltrative nature of the tumor and its involvement of adjacent intestinal structures. Additional findings included inflammatory changes accompanied by secondary degeneration and fibrinopurulent peritonitis. The colonic mucosa demonstrates focal erosive and ulcerative changes.

### 2.5. Postoperative Course

The postoperative course was uneventful. The patient received standard postoperative care, including analgesia, antibiotic therapy, anticoagulation, and intravenous infusion therapy. The patient’s overall condition remained satisfactory throughout the hospital stay. The laparotomy wound healed by primary intention without signs of inflammation. Laboratory parameters remained stable and within normal ranges, and no postoperative complications were observed. The patient was discharged in satisfactory condition with recommendations for outpatient follow-up by an oncologist and a surgeon. Subsequently, the patient has been under outpatient oncologic follow-up. During the 6-month follow-up period, no evidence of tumor recurrence was detected.

## 3. Discussion

Gastrointestinal stromal tumors (GISTs) are rare mesenchymal neoplasms, with an estimated annual incidence of 10–15 cases per million population. They occur predominantly in the stomach and small intestine, whereas colorectal localization is uncommon and cecal involvement is exceedingly rare [4,7]. The stomach (55.6%) and small intestine (31.8%) represent the most frequent sites of GIST occurrence, while the colon (6.0%) and esophagus (0.7%) are rare localizations [8,9]. Within the colon, the sigmoid colon is most commonly affected, followed by the transverse, descending, ascending colon, and cecum [10].

Cecal gastrointestinal stromal tumors represent a rare subset of GISTs. They may occasionally present as ileocolic intussusception in adults, posing a diagnostic challenge due to nonspecific clinical features. In some cases, adult intussusception may be the initial manifestation of an underlying malignancy such as GIST [11,12]. Tumor localization in the right iliac region may mimic appendicitis or an inflammatory infiltrate, often leading to an incorrect initial diagnosis [9,10,13]. In this case, a 39-year-old patient was admitted with a clinical presentation consistent with an appendiceal inflammatory mass with suspected abscess formation. The patient presented with right iliac fossa pain accompanied by systemic inflammatory manifestations, with laboratory findings consistent with a systemic inflammatory response.

Physical examination revealed localized tenderness in the right iliac region with positive rebound tenderness, suggestive of an acute surgical abdomen. Imaging studies, including abdominal CT and ultrasonography, identified an inflammatory mass in the right iliac fossa with surrounding fat stranding and heterogeneous echostructure, initially interpreted as an appendiceal infiltrate with suspected abscess formation.

Despite prior conservative management, the patient demonstrated progressive clinical deterioration and progression of symptoms, indicating failure of non-operative treatment and possible development of purulent complications. Under these circumstances, continued conservative therapy was considered inappropriate, because of the risk of further progression of clinical worsening and potential generalized peritonitis.

Intraoperatively, partial intestinal obstruction was identified, along with secondary phlegmonous appendicitis associated with a cecal tumor of uncertain etiology.

Importantly, no preoperative suspicion of malignancy, including GIST, was established because the clinical and radiological findings were nonspecific and predominantly suggestive of an inflammatory process. In addition, the urgent nature of the presentation precluded extended oncological evaluation, including tumor marker assessment. Thus, emergency surgical intervention was justified by the acute inflammatory process, suspected abscess formation, suspected acute surgical pathology of the abdominal cavity and failure of conservative management, while the diagnosis of an underlying neoplastic process was only made intraoperatively.

This case also highlights the limitations of conventional imaging modalities in diagnosing rare cecal GISTs. Both CT and ultrasonography demonstrated nonspecific inflammatory changes and failed to reliably differentiate between inflammatory infiltrate and neoplastic disease, consistent with previous reports describing the low specificity of imaging in rare colonic GIST localizations [12,13,14]. Definitive diagnosis was established only after surgical resection and histopathological examination. Histopathological confirmation of GIST relies on characteristic morphologic features and immunohistochemical markers, including CD117 (KIT) and DOG1 [15,16]. Molecular alterations involving KIT or PDGFRA genes are of major clinical importance because they determine responsiveness to tyrosine kinase inhibitor therapy [15,16].

This case illustrates a rare presentation of cecal GIST and highlights the difficulty of detecting the tumor even during the initial surgical intervention, as well as the subsequent diagnostic difficulties that ultimately resulted in complications, including partial intestinal obstruction and secondary phlegmonous appendicitis. This reflects an important practical challenge in emergency abdominal surgery, where prior diagnoses may unintentionally bias subsequent clinical assessment. Emergency surgical intervention became unavoidable because of progressive inflammatory symptoms, failure of conservative management, suspected purulent complications, and signs of acute surgical abdominal pathology. Intraoperatively, partial intestinal obstruction and secondary phlegmonous appendicitis associated with a cecal tumor were identified. Rapid emergency surgical intervention and accurate intraoperative identification of the source of the acute abdominal pathology at our clinic were crucial for preventing severe complications.

A particularly important aspect of this case was the persistent absence of oncologic suspicion throughout multiple stages of patient management, including repeated hospitalizations and a prior surgical intervention. During the initial operation at the district hospital, the lesion was interpreted as an appendiceal inflammatory infiltrate, and drainage was performed without identification of the underlying tumor. This initial diagnostic misinterpretation subsequently influenced further clinical decision-making and contributed to repeated attribution of the patient’s symptoms to inflammatory pathology rather than neoplastic disease. At the regional hospital stage, right-sided hydronephrosis was attributed to ureteral compression caused by the presumed appendiceal inflammatory infiltrate. In fact, the tumor-associated inflammatory infiltrate compressed the right ureter, resulting in secondary right-sided hydronephrosis.

However, the present case is distinguished by the unusual combination of partial intestinal obstruction and secondary phlegmonous appendicitis associated with cecal GIST. To the best of our knowledge, this combination has rarely been described in the literature. Complete surgical resection with negative margins remains the cornerstone of treatment and is associated with favorable long-term survival outcomes [4,6,8,11,14,15,16,17]. In the present case, radical tumor resection with primary restoration of intestinal continuity using a side-to-side ileo-ascending anastomosis resulted in a favorable postoperative outcome. The favorable postoperative outcome confirms the appropriateness of the chosen strategy and is consistent with previous studies demonstrating improved long-term outcomes following complete tumor resection [18,19,20,21,22].

This case highlights several important clinical and educational considerations:

- Cecal GIST may clinically and radiologically mimic appendiceal inflammatory infiltrate or abscess formation.
- Repeated diagnostic misinterpretation may occur when oncologic suspicion is absent.
- Rare neoplasms should be considered in patients with atypical, recurrent, or prolonged inflammatory masses of the right iliac fossa.
- Definitive diagnosis may become possible only after emergency surgical intervention and histopathological examination.

Several cases of cecal GIST misdiagnosed as appendiceal infiltrate have previously been reported in the literature [9,10,11,12]. However, the uniqueness of the present case lies in the fact that the patient ultimately underwent emergency surgery for complications of the underlying disease, namely partial intestinal obstruction and secondary phlegmonous appendicitis, which enabled timely radical surgical treatment of the cecal GIST. It is noted that the principal limitation of this report is its single-case nature, which precludes generalization of the findings to a broader patient population. Such cases in the literature are more commonly described in the stomach, whereas those involving the cecum remain rare [23,24]. Nevertheless, given the rarity of cecal GIST and its unusual presentation, this case contributes valuable clinical experience to the existing literature.

## 4. Conclusions

Cecal GIST represents a rare diagnostic challenge because its clinical and radiological presentation may closely mimic appendiceal inflammatory infiltrate or abscess formation. This case illustrates the difficulty of recognizing rare cecal GIST in the absence of oncologic suspicion, even after repeated hospitalizations, imaging studies, and prior surgical intervention.

Conventional imaging modalities may be insufficient for reliable differential diagnosis, and definitive diagnosis often becomes possible only after surgical resection and histopathological evaluation. Emergency surgical intervention may be required because of complications such as intestinal obstruction, secondary appendicitis, or progressive inflammatory changes.

This case also demonstrates the potential for repeated diagnostic misinterpretation across multiple stages of care and highlights the importance of maintaining diagnostic vigilance in patients with atypical or recurrent inflammatory masses of the right iliac fossa. Radical surgical resection with restoration of intestinal continuity remains the cornerstone of treatment and may provide favorable clinical outcomes.

Further accumulation of clinical experience with rare cecal GISTs is necessary to improve diagnostic algorithms and optimize surgical management strategies.

## Figures and Tables

**Figure 1:**
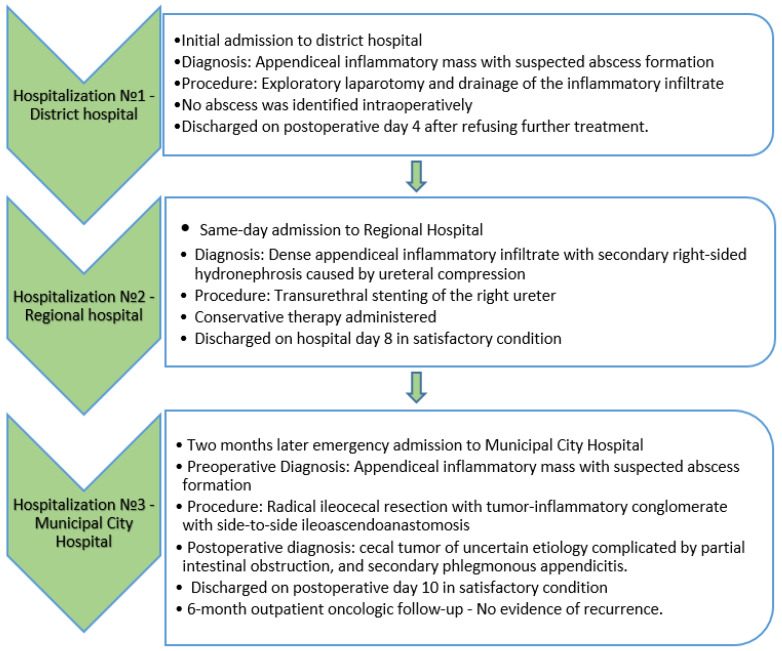
Schematic representation of the patient’s clinical course and treatment timeline.

**Figure 2:**
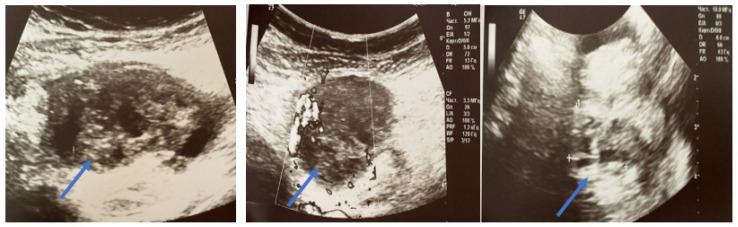
Abdominal ultrasonography. Abdominal ultrasonography demonstrating a heterogeneous inflammatory mass in the right iliac region (blue arrow) with suspected abscess formation.

**Figure 3:**
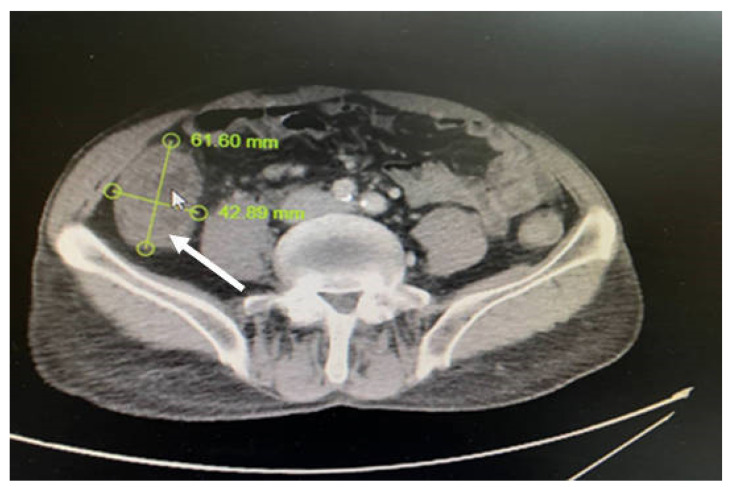
CT scan of the abdominal cavity. Computed tomography (CT) of the abdomen demonstrating the pathological inflammatory mass located in the right iliac region (white arrow) with infiltration of the surrounding adipose tissue.

**Figure 4:**
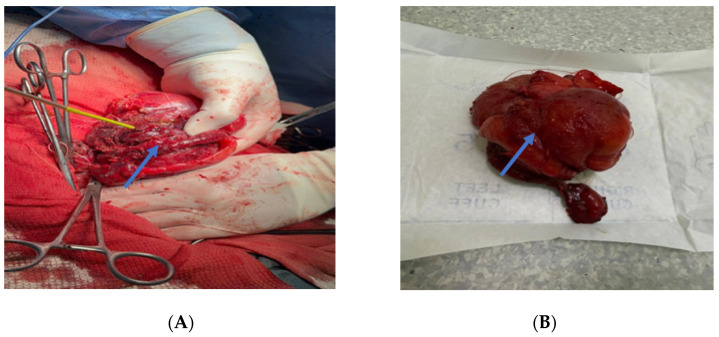
Intraoperative view (**A**) and gross specimen of the cecal tumor (**B**). The gross specimen demonstrates a tumor-like mass emanating from the cecal wall, measuring 8.0 × 5.0 cm, with a dense consistency and a distinctly granular structure (blue arrow). On cut section, the lesion shows a heterogeneous structure, with areas of necrosis and small hemorrhages (blue arrow).

**Figure 5:**
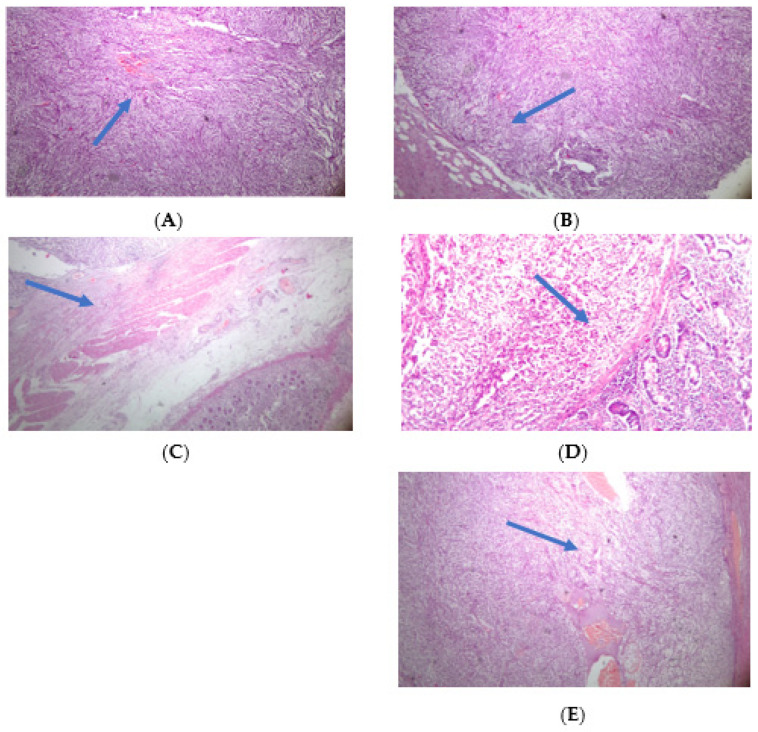
Histological examination of the resected gastrointestinal stromal tumor specimen. Hematoxylin and eosin (H&E) stain, ×5 magnification. (**A**) Tumor section showing intersecting fascicles of spindle-shaped neoplastic cells with small nodular structures composed of epithelioid-type cells (blue arrow). (**B**) Tumor infiltration into the surrounding adipose tissue (blue arrow). (**C**) Tumor infiltration of the muscular layer of the small intestinal wall (blue arrow). (**D**) Tumor infiltration of the submucosal layer of the small intestinal wall (blue arrow). (**E**) Tumor infiltration of the intermuscular layer of the small intestinal wall (blue arrow).

**Table 1:** Temporal changes in the patient’s laboratory findings at admission, during the postoperative period, and at discharge.

Parameters	First Day—At Admission	Second Day	Third Day	Sixth Day	Tenth Day—At Discharge
Blood Biochemistry					
Albumin, g/L	–	29.8	32.5	30.8	32.1
Total protein, g/L	–	48.3	50.9	49.9	51.8
ALT, U/L	–	14.18	10.94	8.95	37.99
AST, U/L	–	10.06	18.00	10.14	22.43
Creatinine, μmol/L	93.69	62.30	99.89	59.64	44.32
Urea, mmol/L	4.47	4.07	5.77	5.27	2.59
Total bilirubin, μmol/L	14.0	9.7	2.5	4.1	3.9
Direct bilirubin, μmol/L	5.9	5.6	2.1	2.3	2.8
Glucose, mmol/L	6.31	8.64	7.11	5.27	5.17
Potassium, mmol/L	4.3	4.3	4.6	4.6	4.3
Sodium, mmol/L	134	136	147	137	135
Total amylase, U/L	43.02	17.78	33.27	45.29	52.73
C-reactive protein, mg/L	22.6	–	–	–	17.6
Coagulation Profile					
APTT, s	33.2	–	–	30.2	–
INR	1.15	–	–	1.15	–
Prothrombin time, s	15.8	–	–	14.4	–
Fibrinogen, g/L	3.8	–	–	3.6	–
Complete Blood Count					
Hemoglobin, g/L	131	108	97	94	97
Hematocrit, %	40.0	32.8	30.8	29.5	30.5
Erythrocytes, ×10^12^/L	4.42	3.60	3.31	3.26	3.29
Leukocytes, ×10^9^/L	12.3	13.5	13.7	11.2	10.8
Neutrophils, %	79.4	81.8	81.9	73.5	75.9
Lymphocytes, %	11.9	7.9	8.6	14.9	13.8
Platelets, ×10^9^/L	242	257	386	451	492
ESR, mm/h	48	49	22	9	8
Urinalysis					
Protein, g/L	0.033	0.033	0.33	–	0.033
Leukocytes	2–4 per field of view	none	none	–	none
Erythrocytes	2–3 per field of view	none	none	–	none
Bacteria	+	–	–	–	-
pH	5.0	5.0	5.0	–	5.0
Specific gravity	1015	1015	1015	–	1015

## Data Availability

The original contributions presented in this study are included in the article. Further inquiries can be directed to the corresponding authors.

## Abbreviations

The following abbreviations are used in this manuscript:

GISTs	Gastrointestinal stromal tumors
CT	Computed tomography
TKIs	Tyrosine kinase inhibitors

## References

[1] Mantese G. (2019). Gastrointestinal stromal tumor: Epidemiology, diagnosis, and treatment. Curr. Opin. Gastroenterol..

[2] Casali P.G., Blay J.Y. (2022). Gastrointestinal stromal tumors: ESMO-EURACAN Clinical Practice Guidelines for diagnosis, treatment, and follow-up. Ann. Oncol..

[3] Joensuu H. (2008). Risk stratification of patients diagnosed with gastrointestinal stromal tumor. Hum. Pathol..

[4] DeMatteo R.P., Lewis J.J., Leung D., Mudan S.S., Woodruff J.M., Brennan M.F. (2000). Two hundred gastrointestinal stromal tumors: Recurrence patterns and prognostic factors for survival. Ann. Surg..

[5] Brčić I., Argyropoulos A., Liegl-Atzwanger B. (2021). Update on Molecular Genetics of Gastrointestinal Stromal Tumors. Diagnostics.

[6] Nishida T., Hirota S., Yanagisawa A., Sugino Y., Minami M., Yamamura Y., Otani Y., Shimada Y., Takahashi F., Kubota T. (2008). Clinical practice guidelines for gastrointestinal stromal tumor (GIST) in Japan: English version. Int. J. Clin. Oncol..

[7] Yu Y., Yu M., Luo L., Zhang Z., Zeng H., Chen Y., Lin Z., Chen M., Wang W. (2024). Molecular characteristics and immune microenvironment of gastrointestinal stromal tumours: Targets for therapeutic strategies. Front. Oncol..

[8] Miettinen M., Sarlomo-Rikala M., Sobin L.H., Lasota J. (2000). Gastrointestinal Stromal Tumors and Leiomyosarcomas in the Colon. Am. J. Surg. Pathol..

[9] Søreide K., Sandvik O.M., Søreide J.A., Giljaca V., Jureckova A., Bulusu V.R. (2016). Global epidemiology of gastrointestinal stromal tumours (GIST): A systematic review of population-based cohort studies. Cancer Epidemiol..

[10] Feng F., Tian Y., Liu Z., Xu G., Liu S., Guo M., Lian X., Fan D., Zhang H. (2016). Clinicopathological features and prognosis of colonic gastrointestinal stromal tumors: Evaluation of a pooled case series. Oncotarget.

[11] Shrestha B.M., Shrestha S., Kharel S., Adhikari S., Tiwari S.B., Kandel B.P., Lakhey P.J. (2021). Cecal gastrointestinal stromal tumor causing ileocolic intussusception in an adult: A rare case report. Int. J. Surg. Case Rep..

[12] Randhawa M.A., Kaynat S., Niaz H., Shahzad T. (2018). Intraluminal Gist, A Rare Cause of Ileo-Ileal Intussusseption in an Elderly Male. Arch. Clin. Biomed. Res..

[13] Chun J.M., Lim K.H. (2016). Gastrointestinal stromal tumor of the vermiform appendix mimicking Meckel’s diverticulum: Case report with literature review. Int. J. Surg. Case Rep..

[14] Lopes J.M., Carneiro F., Chaves P., Ensari A. (2017). C-Kit (CD117), Gastrointestinal Stromal Tumors (GISTs). Pathology of the Gastrointestinal Tract, Encyclopedia of Pathology.

[15] Rubin B.P., Singer S., Tsao C., Duensing A., Lux M.L., Ruiz R., Hibbard M.K., Chen C.J., Xiao S., A Tuveson D. (2001). KIT activation is a ubiquitous feature of gastrointestinal stromal tumors. Cancer Res..

[16] Jingnuo D., Huachuan Z., Da Z., Zhi D., Yan C., Fazhi Z. (2026). Gastrointestinal Stromal Tumors: Molecular Mechanisms of Drug Resistance and Advances in Therapeutic Strategies. J. Gastroenterol. Hepatol..

[17] Schaefer I.-M., DeMatteo R.P., Serrano C. (2022). The GIST of Advances in Treatment of Advanced Gastrointestinal Stromal Tumor. Am. Soc. Clin. Oncol. Educ. Book.

[18] Corless C.L., Fletcher J.A., Heinrich M.C. (2004). Biology of GIST. J. Clin. Oncol..

[19] Ahmed M. (2020). Recent advances in the management of gastrointestinal stromal tumor. World J. Clin. Cases.

[20] Beecroft J.R., Brar S., Feng X., Hamilton T., Han-Lee C., Henning J.-W., Josephy P.D., Khalili K., Ko Y.-J., Lemieux C. (2024). Pan-Canadian consensus recommendations for GIST management in high- and low-throughput centres across Canada. Ther. Adv. Med. Oncol..

[21] Jones R.L., Golčić M. (2023). Recent advances in the systemic treatment of gastrointestinal stromal tumors. Cancer Biol. Med..

[22] Golčić M., Jones R.L., Huang P., Napolitano A. (2023). Evaluation of Systemic Treatment Options for Gastrointestinal Stromal Tumours. Cancers.

[23] von Mehren M., Joensuu H. (2018). Gastrointestinal Stromal Tumors. J. Clin. Oncol..

[24] Enodien B., Hendie D., Müller T., Taha-Mehlitz S., Frey D.M., Taha A. (2023). Gastrointestinal Stromal Tumor (GIST): A Remarkable Case Report and Literature Review. Cureus.

